# Characterization of Terpenoids from the Root of *Ceriops tagal* with Antifouling Activity

**DOI:** 10.3390/ijms12106517

**Published:** 2011-10-06

**Authors:** Jun-De Chen, Rui-Zao Yi, Yi-Ming Lin, Dan-Qing Feng, Hai-Chao Zhou, Zhan-Chang Wang

**Affiliations:** 1 Research Center for the Chemistry and Chemical Engineering of Marine Biological Resource, The Third Institute of Oceanography of the State Oceanic Administration, Xiamen 361005, China; E-Mail: yiruizao@163.com; 2 Department of Biology, School of Life Sciences, Xiamen University, Xiamen 361005, China; E-Mails: linym1967@yahoo.com.cn (Y.-M.L.); zhouhc2010@yahoo.com.cn (H.-C.Z.); 3 Department of Oceanography, College of Oceanography and Environmental Science, Xiamen University, Xiamen 361005, China; E-Mails: dqfeng@xmu.edu.cn (D.-Q.F.); wzc_ttt@sina.com.cn

**Keywords:** terpenoids, antifouling activity, barnacle, *Ceriops tagal*, structure-activity relationship

## Abstract

One new dimeric diterpenoid, 8(14)-enyl-pimar-2′(3′)-en-4′(18′)-en-15′(16′)-endolabr- 16,15,2′,3′-oxoan-16-one (**1**) and five known terpenoids: Tagalsin C (**2**), Tagalsin I (**3**), lup-20(29)-ene-3*β*,28-diol (**4**), 3-oxolup-20(29)-en-28-oic acid (**5**) and 28-hydroxylup- 20(29)-en-3-one (**6**) were isolated from the roots of the mangrove plant *Ceriops tagal*. Their structures and relative stereochemistry were elucidated by means of extensive NMR, IR and MS analysis. The antifouling activity against larval settlement of the barnacle *Balanus albicostatus* were evaluated using capsaicin as a positive control. All these terpenoids exhibited antifouling activity against cyprid larvae of the barnacle without significant toxicity. The structure-activity relationship results demonstrated that the order of antifouling activity was diterpenoid (Compound **2**) > triterpenoid (Compounds **4**, **5** and **6**) > dimeric diterpenoid (Compounds **1** and **3**). The functional groups on the C-28 position of lupane triterpenoid significantly affect the antifouling activity. The diterpenoid dimmer with two identical diterpenoid subunits might display more potent antifouling activity than one with two different diterpenoid subunits. The stability test showed that Compounds **2**, **4**, **5** and **6** remained stable over 2-month exposure under filtered seawater.

## 1. Introduction

It is well known that marine fouling organisms, settling on ship hulls and other artificial surfaces submerged in seawaters, cause technical and economic problems [1,2]. Antifouling paints, containing organotin compounds such as tributyltin (TBT) and tributyltin oxide (TBTO), are very effective in controlling these fouling organisms. However, these substances have been found to be toxic to many non-target marine organisms and to pollute the marine ecosystem [2,3]. These have resulted in the total ban of the application of organotin-based antifouling paints in January 2008 in many countries [2]. Currently, some booster biocides are used for antifouling paints, but they may also pollute the aquatic environments [4]. Thus, there is an urgent demand for finding new antifouling agents which are effective and environmentally friendly. In many studies, it is suggested that marine organisms have both physical and chemical methods to protect themselves from the harmful process of biofouling [5–7]. The key chemical antifouling mechanism of marine organisms occurs via the production of secondary metabolites (also known as natural products) which deter foulers [2,8–14]. Based on recent research, terpenoids, which are an important natural product, may act as defensive compounds for a variety of organisms in the marine world [15,16]. *Ceriop tagal*, a mangrove plant species in the Family of Rhizophoraceae, has been proved to possess a series of antifouling terpenoids with unique structural diversity [9,14]. Moreover, our previous work has demonstrated that pimarane diterpenoids isolated from the *C. tagal* can significantly inhibit larval settlement of the barnacle *Balanus albicostatus*, which is one important fouling organism in East Asian coastal waters [9]. The structure–activity relationship (SAR) analysis of these pimarane diterpenoids suggested that the functional groups on the C-15 and C-16 position of pimarane diterpenoid were responsible for the antifouling activity [9]. This was further confirmed by the result obtained from our SAR studies on synthetic pimarane diterpenoids [17].

**Figure 1:**
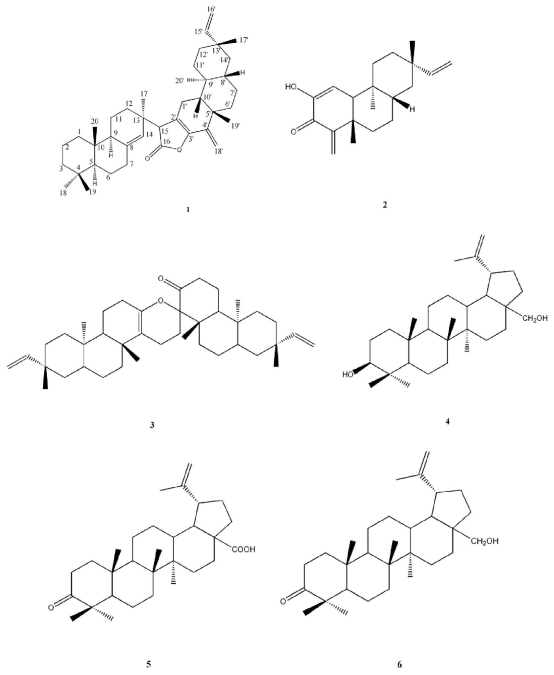
Structures of the isolated terpenoids **1**–**6**.

Due to the highly structural diversity of terpenoids, the structure–activity of mangrove terpenoids merits further investigation. We obtained six terpenoids from *C. tagal*, and then evaluated their antifouling activity against larval settlement of the barnacle *B. albicostatus* using capsaicin as a positive control. These antifouling terpenoids included one new dimeric diterpenoid, 8(14)-enyl-pimar- 2′(3′)-en-4′(18′)-en-15′(16′)-en-dolabr-16,15,2′,3′-oxoan-16-one (**1**) and five known terpenoids, designated as Tagalsin C (**2**), Tagalsin I (**3**), lup-20(29)-ene-3*β*,28-diol (**4**), 3-oxolup-20(29)-en-28-oic acid (**5**) and 28-hydroxylup-20(29)-en-3-one (**6**) (Figure 1). The isolation, structural elucidation and antifouling activity of these compounds are described in the present study.

## 2. Results and Discussion

### 2.1. Structural Elucidation of Terpenoids

Compound **1** was obtained as yellow oil, and its molecular composition of C_40_H_58_O_2_ was established by HR-ESI-MS (*m/z* 571.4751 [M + H+]^+^, C_40_H_58_O_2_^+^). The ESI-MS of **1** showed quasimolecular ion peaks at *m/z* 571.3 [M + 1]^+^ and 592.7 [M + Na]^+^, respectively. The IR absorptions at 1724 1650, 1237, 1001 and 798 cm^–1^ showed the presence of carbalkoxy and double groups. Analyses of the ^1^H-NMR, ^13^C-NMR, DEPT and HMQC spectra revealed not only the presence of 7 methyls, 15 methylenes, 7 methines and 11 quaternary carbon (including one carbalkoxy group at δ_C_ 177.0, d), but also the presence of one tetrasubstituted double bond, one tetrasubstituted double bond and two terminal double bonds (Tables 1). Four methyl singlets (δ_C_ 24.7, q; 33.7, q; 22.1, q; 15.4, q) and one trisubstituted double bond (δ_C_ 137.7, s; 128.5, d) suggesting that a pimarane-type diterpenoid structure might be presented [9,18–21]. Meanwhile, three methyl singlets (δ_C_ 23.0, q; 33.1, q; 11.8, q) and two terminal double bonds (δ_C_ 139.5, s; 105.9, t; 151.0, d; 109.0, t) might indicate the structure of a dolabradiene-type diterpenoid [22–25]. Therefore, Compound **1** seemed to be a dimeric diterpenoid composed of two moieties, one being a pimarane-type diterpenoid (part A) and the other a dolabradiene-type diterpenoid (part B).

**Figure 2:**
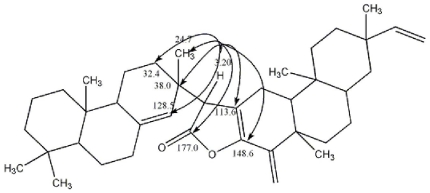
Key HMBC of Compound 1.

The HMBC correlations of Me18 (δ_H_ 0.89, s) with C-5 (δ_C_ 55.0, d), C-3 (δ_C_ 42.1, t), C-4 (δ_C_ 33.3, s) and C-19 (δ_C_ 22.1, q); Me19 (δ_H_ 0.85, s) with C-5 (δ_C_ 55.0, d), C-3 (δ_C_ 42.1, t), C-4 (δ_C_ 33.3, s) and C-18 (δ_C_ 33.7, q); Me20 (δ_H_ 0.82, s) with C-9 (δ_C_ 49.8, d), C-1 (δ_C_ 39.2, t); Me17 (δ_H_ 0.99, s) with C-14 (δ_C_ 128.5, d), C-15 (δ_C_ 56.2, d), C-13 (δ_C_ 38.0, s) and C-12 (δ_C_ 32.4, t); H-5 (δ_H_ 1.06, m) with C-20 (δ_C_ 15.4, q), C-6 (δ_C_ 22.7, t); H-9 (δ_H_ 1.74, m) with C-8 (δ_C_ 137.7, s), C-14 (δ_C_ 128.5, d), C-20 (δ_C_ 15.4, q), C-11 (δ_C_ 18.6, t)and C-10 (δ_C_ 38.87, s); H-14 (δ_H_ 5.55, s) with C-9 (δ_C_ 49.8, d), C-13 (δ_C_ 38.0, s), C-7 (δ_C_ 36.23, t), C-12 (δ_C_ 32.4, t) and C-17 (δ_C_ 24.7, q) confirmed that part A belonged to a pimarane-type diterpenoid. The HMBC correlations of Me-20′ (δ_H_ 0.57, s) with C-10′ (δ_C_ 53.3, d), C-8′ (δ_C_ 43.0, d), C-9′ (δ_C_ 38.1, s ) and C-11′ (δ_C_ 35.2, t); Me-19′ (δ_H_ 1.14, s) with C-4′ (δ_C_ 139.5, s), C-10′ (δ_C_ 53.3, d), C-5′ (δ_C_ 39.1, s ) and C-6′ (δ_C_ 36.8, t); Me-17′ (δ_H_ 1.01, s) with C-15′ (δ_C_ 151.0, d), C-13′ (δ_C_ 36.18, s) and C-12′ (δ_C_ 32.0, t); H-15′ (δ_H_ 5.81, dd, *J* = 12.6, 21 Hz) with C-14′ (δ_C_ 38.93, t), C-13′ (δ_C_ 36.18, s), C-12′ (δ_C_ 32.0, t) and C-17′ (δ_C_ 23.0, q); H-16′ (δ_H_ 4.82, d, *J* = 13.2 Hz) with C-15′ (δ_C_ 151.0, d) and C-13′ (δ_C_ 36.18, s); H-1′ (δ_H_ 2.28, m) with C-3′ (δ_C_ 148.6, s), C-2′ (δ_C_ 113.6, s), C-10′ (δ_C_ 53.3, d), C-9′ (δ_C_ 38.1, s) and C-5′ (δ_C_ 39.1, s); H-1′ (δ_H_ 2.56, dd, *J* = 7.8, 14.4 Hz) with C-3′ (δ_C_ 148.6, s), C-2′ (δ_C_ 113.6, s), C-10′ (δ_C_ 53.3, d) and C-9′ (δ_C_ 38.1, s); H-10′ (δ_H_ 1.38, m) with C-5′ (*δ*_C_ 39.1, s), C-9′ (*δ*_C_ 38.1, s), C-11′ (*δ*_C_ 35.2, t), C-19′ (*δ*_C_ 33.1, q) and C-1′ (*δ*_C_ 22.2, t); H-18′ (*δ*_H_ 5.26, s) with C-5′ (δ_C_ 39.1, s), C-3′ (δ_C_ 148.6, s) and C-4′ (δ_C_ 139.5, s); H-18′ (δ_H_ 5.08, s) with C-5′ (δ_C_ 39.1, s), C-3′ (δ_C_ 148.6, s) and C-4′ (δ_C_ 139.5, s) confirmed that part B belonged to a dolabradiene-type diterpenoid. Then the chemical shift of δ_C_ 56.2 (d, C-15), δ_C_ 177.0 (s, C-16), δ_C_ 148.6 (s, C-3′) were compatible with a bond between C-3′ and the lactone O-atom, which was further confirmed by the HR-ESI-MS spectrum. The HMBC correlations from H-15 (δ_H_ 3.20, s) to C-3′ (δ_C_ 148.6, s), C-14 (δ_C_ 128.5, d), C-2′ (δ_C_ 113.6, s), C-13 (δ_C_ 38.0, s), C-12 (δ_C_ 32.4, t), C-17 (δ_C_ 24.7, q) and C-16 (δ_C_ 177.0, s) established the connection between part A and part B at the site of C-15, C-2′ and C-3′ (Figure 2). Furthermore, other HMBC correlations along with the comparison of its NMR data and the known pimarane-type diterpenoid and dolabradiene-type diterpenoid comprehensively confirmed that the planar structure of **1** was assigned as a novel dimeric diterpenoid [18–26].

**Figure 3:**
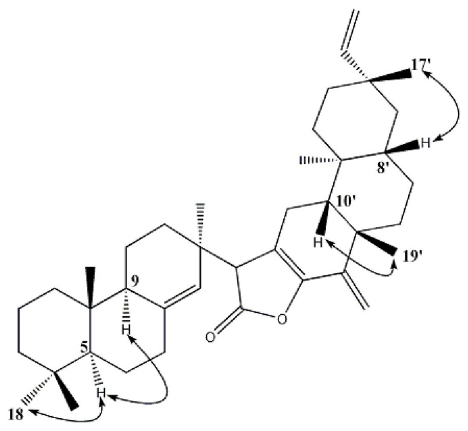
Key NOE correlations of Compound 1.

The relative stereochemistry of **1** was established by NOE spectrum. Comparison of the NMR data of **1** with the pimarane-type diterpenoid and dolabradiene-type diterpenoid showed that the Me-17 (δ_C_ 24.7, q), Me-18 (δ_C_ 33.7, q) and Me-20′ (δ_C_ 11.8, q) were in a relative α-orientation, while Me-19 (δ_C_ 22.1, q), Me-20 (δ_C_ 15.4, q), Me-19′ (δ_C_ 33.1, q) and Me-17′ (δ_C_ 23.0, q) were in a relative *β*-orientation [18–26]. NOE correlations between Me-18/H-5 and H-5/H-9 indicated that H-5 and H-9 were on the β-side of **1**. NOE correlations between H-10′/Me-19′ and Me-17′/H-8′ indicated that they were oriented on another side of the nucleus (Figure 3).

Five known terpenoids, Tagalsin C (**2**), Tagalsin I (**3**), lup-20(29)-ene-3*β*,28-diol (**4**), 3-oxolup- 20(29)-en-28-oic acid (**5**) and 28-hydroxylup-20(29)-en-3-one (**6**), were identified by comparison of their physical and spectral data with those in the literature [23,27–31].

### 2.2. Antifouling Activity of Terpenoids

**Figure 4:**
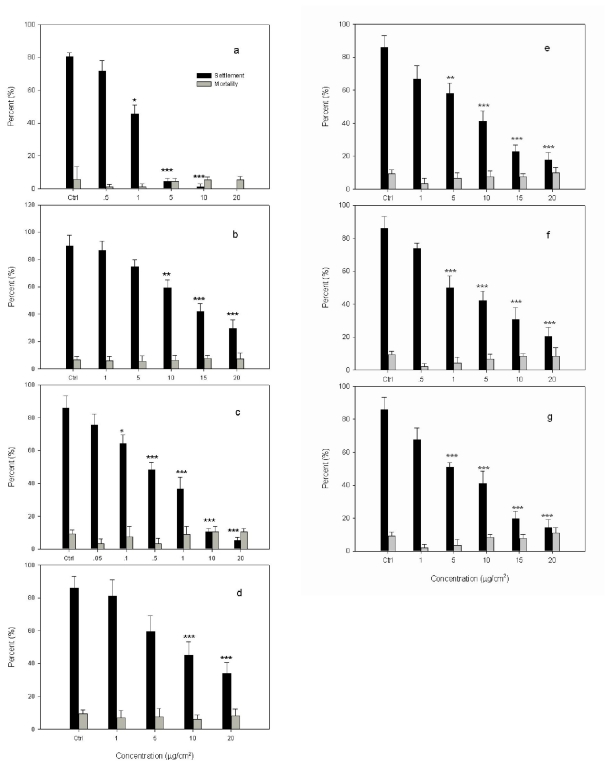
Effects of compounds on settlement and survival of *B. albicostatus* cyprids. **a**: Capsaicin; **b**: 8(14)-enyl-pimar-2′(3′)-en-4′(18′)-en-15′(16′)-en-dolabr-16,15,2′,3′-oxoan- 16-one (**1**); **c**: Tagalsin C (**2**); **d**: Tagalsin I (**3**); **e**: lup-20(29)-ene-3*β*,28-diol (**4**); **f**: 3-oxolup-20(29)-en-28-oic acid (**5**); **g**: 28-hydroxylup-20(29)-en-3-one (**6**). Data were analyzed using one-way ANOVA, where *P < 0.05; **P < 0.01 and ***P < 0.001 were significantly different from the negative control.

The effects of terpenoids on settlement and mortality of *B. albicostatus* cyprids are shown in Figure 4 and their EC_50_ and LC_50_ values are summarized in Table 2. Capsaicin was used as a positive standard with EC_50_ value of 1.32 μg/cm^2^ (Figure 4 and Table 2). Filtered seawater (FSW) was used as a negative control. Comparison of means using ANOVA and Dunnet’s test showed that all of the terpenoids significantly inhibited settlement compared with the negative control (*P* < 0.001) without significant toxicity (*P* > 0.05). From Dunnet’s test, capsaicin significantly reduced larval settlement at 5 μg/cm^2^ (*P* < 0.001), and Compounds **1**–**6** significantly reduced larval settlement at 0.5–15 μg/cm^2^ (*P* < 0.001). Moreover, the non-toxicity compounds were defined as those that do not directly kill fouling organisms at or near the levels at which they deter fouling [32]. In the present study, the LC_50_ values of these terpenoids were all above 250 μg/cm^2^. The lower EC_50_/LC_50_ ratio indicated that all these terpenoids had the ability to inhibit larval settlement in a non-toxic way. In addition, the stability test showed that Compounds **1** and **3** were degraded while Compounds **2**, **4**, **5** and **6** remained stable over 2-month exposure under FSW.

As shown in Table 2, Compounds **1**–**6** exhibited antifouling activity against cyprid larvae of the barnacle *B. albicostatus*, with EC_50_ values ranging from 0.65 to 13.9 μg/cm^2^. Compound **2** was more active than capsaicin, while Compounds **1**, **3**, **4**, **5** and **6** showed antifouling activity weaker than capsaicin. Among these terpenoids, diterpenoid (Compound **2**) showed the best antifouling activity against cyprid larvae of the barnacle *B. albicostatus*, followed by triterpenoid (Compounds **4**, **5** and **6**), and then dimeric diterpenoid (Compounds **1** and **3**). In addition, the antifouling activity of Compound **3** was stronger than Compound **1**, which suggested that the diterpenoid dimmer with two identical diterpenoid subunits might display more potent antifouling activity than the one with two different diterpenoid subunits. By comparing the EC_50_ value of Compound **5** and Compound **6** and considering that the only difference between the structures of these two compounds is the difference group on the C-28 of lupane triterpenoid, it is evident that the carboxyl group at the C-28 of lupane triterpenoid might have a more potent antifouling activity than the oxygenated methylene group. However, when carbonyl group at the C-3 position in lupane triterpenoid (Compound **6**) is substituted by hydroxyl group, the resulting compound (Compound **4**) showed almost the same antifouling activity as Compound **6**. This result indicated that the hydroxyl group might be another important functional group expressing potent antifouling activity in lupane triterpenoid.

## 3. Experimental Section

### 3.1. General

^1^H-NMR spectra were measured with Varian UNITY PLUS 500 spectrometer (USA). ^1^H-, ^13^C- and 2D-NMR spectra were measured with Varian INOVA 600 spectrometer (USA). High-resolution ESI mass spectra data were measured with a Waters Q-TOF MicroTM spectrometer (USA) and were provided by Zhengzhou University, China. Low-resolution ESI mass spectra data were recorded on an AB 3200Q TRAP spectrometer (USA). IR spectra were measured using a Nicolet 380 FT-IR spectrophotometer (USA). The optical rotation data were obtained on a Rudolph Autopol IV polarimeter (USA). 200–300 mesh Silica gel (Qingdao Marine Chemical Factory, Qingdao, China) were used for column chromatography. Compounds were monitored by TLC on GF 254 silica gel (Qingdao Marine Chemical Factory, Qingdao, China). ODS and Sephadex LH-20 were obtained from Pharmacia Co. Capsaicin was purchased from Sigma Chemical Co., USA, with purity above 95%.

### 3.2. Extraction and Isolation

The roots of *C. tagal* were collected from Hainan Province, China in July 2005. The air-dried and powdered material (4.1 kg) was extracted with 95% EtOH at room temperature. The extract was concentrated and partitioned sequentially with petroleum ether, ethyl acetate and H_2_O. The petroleum ether extract (11.5 g) was subjected to a silica gel column, eluted with a petroleum ether–ethyl acetate gradient to obtain 13 fractions (A–M). Fraction C (0.135 g) was chromatographed over silica gel and Sephadex LH-20 repeatedly, and the major fraction obtained was then purified by reversed-phase semi-preparation HPLC (elution with 1:1 hexane-CHCl_3_) to yield Compound **1** (50 mg). Fraction K (1.059 g) was chromatographed over silica gel and Sephadex LH-20 repeatedly (elution with 30:1 petroleum ether–acetone) to yield Compound **4** (5 mg) and Compound **5** (10 mg). Fraction J (5.3 g) was chromatographed over silica gel repeatedly (elution with 30:1 petroleum ether–acetone) to yield Compound **6** (6 mg). Moreover, the ethyl acetate extract (73.6 g) was subjected to a silica gel column, eluted with a CHCl_3_–MeOH gradient to obtain 12 fractions (A2-L2). Fraction B2 (8.32 g) was chromatographed over silica gel and Sephadex LH-20 repeatedly. Compound **2** (10 mg) was further separated by silica gel (elution with 40:1 petroleum ether–ethyl acetate) and Compound **3** (53 mg) was purified by silica gel (elution with 2.5:1 hexane-CHCl_3_).

*8(14)-enyl-pimar-2′(3′)-en-4′(18′)-en-15′(16′)-en-dolabr-16,15,2′,3′-oxoan-16-one* (**1**): C_40_H_58_O_2_; yellow oil; [α]_D_^25^ = +68.25 (*c* = 0.50, CHCl_3_); UV (CHCl_3_): 214 (2.5905), 224 (3.0367), 234 (2.929), 244 (2.7263). IR *ν*_max_ (KBr): 1724,1650, 1237, 1001, 798 cm^–1^; ESI-MS, *m/z* 571.3 ([M + 1]^+^), 592.7 [M + Na]^+^, HR-ESI-MS at *m/z* 571.4751 ([M + H+]^+^, C_40_H_58_O_2_^+^calc. 571.4741 ); ^1^H-NMR and ^13^C-NMR data for **1** are listed in Table 1.

### 3.3. Antifouling Assay

Antifouling efficacies of the six terpenoids isolated from *C. tagal* were evaluated by the settlement inhibition assay with cyprid larvae of *B. albicostatus. B. albicostatus* adults were collected from intertidal rocks in Xiamen, China. Adults released the nauplius I and nauplius II stages upon immersion in seawater after dried for 12 h [8]. Naupilar larvae actively swimming towards the light were collected using a pipette. They were cultured in filtered seawater (FSW, 0.22 μm, salinity 30‰, and temperature 25 °C) by feeding with the diatom Chaetoceros muelleri at a concentration of 2.0 × 10^5^ cells·mL^−1^. The FSW and diet were renewed everyday. After 5~6 days, most of the nauplii had metamorphosed to cyprids, and then the cyprids were collected within 24 h. Antifouling efficacies of the six terpenoids were investigated according to the method of Hellio *et al*. with a minor modification [33]. The tested compounds were introduced to the glass dishes using CH_2_Cl_2_ as carrier solvent. After evaporation of the solvent at room temperature, 30 cyprids and 10 mL FSW were added to each glass dishe. There were three replicates for FSW negative control and each concentration of the tested compounds. Dishes were incubated in dark at 25 °C for 48 h. After that, numbers of larvae which settled, died or did not settle in each replicate were enumerated under a stereomicroscope. Cyprids that did not move and did not respond to a touch with a metal probe were regarded as dead [34]. Cyprids that permanently attached and metamorphosed were counted as settled [33,35]. In addition, the stability of terpenoids was tested using the method of thin layer chromatography (TLC), which were identified by comparison of the spots and *R*_f_ values of these terpenoids before and after 2-month exposure in FSW control [17]. Three replicates were set up for each of FSW control. The tested terpenoids were dissolved in CH_2_Cl_2_ and evaporated after 2-month exposure in FSW (three replicates). Compounds that showed the same spots and *R*_f_ values with before were regarded as stability. Compounds that showed the different spots and *R*_f_ values with before were regarded as degradation. Moreover, statistical calculations were performed with the SPSS software package. One-way ANOVA followed by a Dunnett's test for multiple comparisons of treatment means with a negative control was used for the settlement or mortality percentages analysis. The significance level was defined as *p* < 0.05. Both EC_50_ (the concentration that reduces the settlement rate by 50% relative to the negative control) and LC_50_ (the concentration that results in 50% mortality relative to the negative control) were estimated using the Spearman-Karber method with a 95% confidence interval [36,37].

## 4. Conclusions

One new dimeric diterpenoid and five known terpenoids were isolated from the roots of *C. tagal*. These terpenoids were able to inhibit cyprid settlement (EC_50_ values range of 0.65 to 13.9 μg/cm^2^) without significant toxicity. Moreover, the functional groups on the C-28 position of lupane triterpenoid significantly affect their antifouling activity. The diterpenoid dimmer with two identical diterpenoid subunits might display more potent antifouling activity than the one with two different diterpenoid subunits. Diterpenoid showed the strongest antifouling activity against cyprid larvae of the barnacle *B. albicostatus* followed by triterpenoid, and then dimeric diterpenoid. The stability test showed that Compounds **2**, **4**, **5** and **6** remained stable over a 2-month exposure under FSW.

## Figures and Tables

**Table 1:** ^1^H-NMR Data (500 MHz) and ^13^C-NMR Data (125 MHz) for Compound **1** (CDCl_3_).

No.	δ_C_	δ_H_	No.	δ_C_	δ_H_
1	39.2 t	0.95 (m)	1′	22.2 t	2.28 (m)
		1.71 (m)			2.56 (dd, *J* = 7.8, 14.4 Hz)
2	19.0 t	1.53 (m)	2′	113.6 s	
3	42.1 t	1.40 (m)	3′	148.6 s	
		1.19 (m)	4′	139.5 s	
4	33.3 s		5′	39.1 s	
5	55.0 d	1.06 (m)	6′	36.8 t	2.17 (m)
6	22.7 t	1.58 (m)			1.54 (m)
		1.34 (m)	7′	25.6 t	1.19 (m)
7	36.23 t	2.05 (m)	8′	43.0 d	1.47 (m)
		2.36 (d, *J* = 15 Hz)	9′	38.1 s	
8	137.7 s		10′	53.3 d	1.38 (m)
9	49.8 d	1.74 (m)	11′	35.2 t	1.60 (m)
10	38.87 s				1.34 (m)
11	18.6 t	1.60 (m)	12′	32.0 t	1.54 (m)
		1.10 (m)			1.31 (m)
12	32.4 t	1.68 (m)	13′	36.18 s	
		1.22 (m)	14′	38.93 t	1.36 (m)
13	38.0 s				1.04 (m)
14	128.5 d	5.55 (s)	15′	151.0 d	5.81 (dd, *J* = 12.6, 21 Hz)
15	56.2 d	3.20 (s)	16′	109.0 t	4.82 (d, *J* = 13.2 Hz)
					4.92 (d, *J* = 21 Hz)
16	177.0 s		17′	23.0 q	1.01 (s)
17	24.7 q	0.99 (s)	18′	105.9 t	5.26 (s)
18	33.7 q	0.89 (s)			5.08 (s)
19	22.1 q	0.85 (s)	19′	33.1 q	1.14 (s)
20	15.4 q	0.82 (s)	20′	11.8 q	0.57 (s)

**Table 2:** Antifouling activity of the investigated terpenoids against *B. albicostatus* cyprid larvae.

Compound	Skeleton structure	EC_50_ (μg/cm^2^)	LC_50_ (μg/cm^2^)
Capsaicin (positive control)	-	1.32 ± 0.02 b	> 250
8(14)-enyl-pimar-2′(3′)-en-4′(18′)-en-15′(16′)-en-dolabr-16,15,2′,3′-oxoan-16-one (**1**)	Dimeric diterpenoid	13.94 ± 0.81 f	> 250
Tagalsin C (**2**)	Diterpenoid	0.65 ± 0.02 a	> 250
Tagalsin I (**3**)	Dimeric diterpenoid	11.67 ± 0.47 e	> 250
lup-20(29)-ene-3β,28-diol (**4**)	Triterpenoid	9.27 ± 0.30 d	> 250
3-oxolup-20(29)-en-28-oic acid (**5**)	Triterpenoid	3.50 ± 0.24 c	> 250
28-hydroxylup-20(29)-en-3-one (**6**)	Triterpenoid	8.73 ± 0.43 d	> 250

a–f Values with different letters in the same column of EC_50_ are significantly different at *p* < 0.05 level.
